# Efficient generation of antigen-specific CTLs by the BAFF-activated human B Lymphocytes as APCs: a novel approach for immunotherapy

**DOI:** 10.18632/oncotarget.12792

**Published:** 2016-10-21

**Authors:** Zhang Yiwen, Gao Shilin, Chen Yingshi, Su Lishi, Luo Baohong, Liu Chao, Li Linghua, Pan Ting, Zhang Hui

**Affiliations:** ^1^ Institute of Human Virology, Sun Yat-sen University, Guangzhou, Guangdong, 510080, China; ^2^ Key Laboratory of Tropical Disease Control of Ministry of Education, Sun Yat-sen University, Guangzhou, Guangdong, 510080, China; ^3^ Guangdong Engineering Research Center for Antimicrobial Agent and Immunotechnology, Zhongshan School of Medicine, Sun Yat-sen University, Guangzhou, Guangdong, 510080, China; ^4^ Department of Infectious Diseases, Guangzhou 8th People's Hospital, Guangzhou, Guangdong, 510080, China

**Keywords:** B-lymphocytes, APC, BAFF, HIV-immunotherapy, tumor

## Abstract

Efficient antigen presentation is indispensable for cytotoxic T lymphocyte (CTL)-mediated immunotherapy. B-lymphocytes propagated with CD40L have been developed as antigen-presenting cells (APCs), but this capacity needs further optimization. Here, we aimed to expand human B-lymphocytes on a large scale while maintaining their antigen-presenting ability by using both CD40L and B-cell activating factor (BAFF). The addition of BAFF enhanced the expansion efficiency and prolonged the culture time without causing apoptosis of the expanded B-cells. This method thus provided an almost unlimited source of cellular adjuvant to achieve sufficient expansion of CTLs in cases where several rounds of stimulation are required. We also showed that the addition of BAFF significantly enhanced the expression of major costimulatory molecules, CD80 and CD86. Subsequently, the antigen-presenting ability of the B-lymphocytes also increased. Consequently, these B-lymphocytes showed robust CTL responses to inhibit tumor growth after tumor-specific peptide pulses. A similar method induced potent antigen-specific CTL responses, which effectively eradicated human immunodeficiency virus type 1 (HIV-1) latency in CD4 T-lymphocytes isolated from patients receiving suppressive anti-retroviral therapy (ART). Together, our findings indicate that potent antigen-specific CTLs can be generated using BAFF-activated B-lymphocytes as APCs *ex vivo.* This approach can be applied for CTL-mediated immunotherapy in patients with cancers or chronic viral infections.

## INTRODUCTION

Cytotoxic T lymphocyte (CTL)-mediated immuno-therapy provides a promising approach for the treatment of patients with cancers or chronic viral infections [1–8]. Adoptive T-cell immunotherapy requires the enrichment and expansion of antigen-specific CTLs for infusion, both of which need highly efficient antigen presentation by antigen-presenting cells (APCs). Dendritic cells (DCs) [9, 10], activated macrophages [11], or activated B cells [12–14] are all capable of presenting antigens. Among them, DCs are considered the most efficient APCs for immunotherapy owing to their high efficiency of antigen capture, processing, and migration. However, the low proportion of DCs in the peripheral blood and their poor expansion efficiencies limit their utility as APCs in immunotherapy for the generation of antigen-specific T cells on a large scale [15, 16]. In contrast, B-lymphocytes account for a larger proportion of the peripheral blood mononuclear cells (PMBCs) (10% to 15%) and can undergo efficient expansion *in vitro* [17]. Moreover, B cells appear to have additional unique characteristics such as the ability to induce the proliferation of a significantly higher percentage of T cells and to increase the level of INF-γ without increasing IL-10 production from T cells [17]. B cells can also be efficiently amplified using simple methods and at a low cost *ex vivo* [18]. Considering their capabilities to generate extensive antigen-specific T cells, activated B cells have been identified as an alternative source of APCs for adoptive immunotherapies [19, 20].

Activation and efficient culture of B-lymphocytes *ex vivo* was introduced after the CD40 ligand (CD40L) system was reported [17, 20, 21]. Interaction between CD40L on the surface of a stable 3T3-CD40L cell line and CD40 on B cells is important for the induction of the clonal expansion of B cells *ex vivo* [15, 22]. The CD40L system provides an efficient method for expanding B cells as APCs without the use of viral components such as Epstein-Barr viruses or gene-transfer technology [15, 23]. After co-culture with 3T3-CD40L feeder cells, B cells obtain antigen-presenting ability by increasing the expression of major histocompatibility complex (MHC) class I and class II molecules and by inducing the expression of costimulatory molecules CD80 and CD86 [24]. The antigen-presenting ability of B cells gained importance when their roles in cancer therapies [19, 25, 26] and in priming T-cell responses to viral neoantigens were discovered [15, 24, 27]. However, CD40L can increase apoptosis of human B cells [28–31], which constitutes a significant obstacle for long-term B-cell expansion *in vitro*. Therefore, the method for B cell expansion *in vitro* needs to be optimized to allow their application on a large scale.

BAFF, also named Blys, is a member of the TNF super family and was originally identified as an important factor responsible for B cell survival and maturation [32–34]. BAFF binds to several receptors including Transmembrane activator and CAML interactor (TACI), BAFF receptor (BAFF-R), and B cell maturation antigen (BCMA) [35, 36]. BCMA has been known to promote the antigen-presenting function of B cells and to enhance the survival of long-lived plasma cells (LLPCs) in mouse bone marrow. TACI signaling also plays a role in the BAFF-mediated upregulation of MHC class II expression [37, 38]. BAFF-R appears to be particularly important for the survival and maturation of B cells based on the fact that BAFF-R-deficient mice were found to share a disrupted B cell maturation phenotype similar to that of BAFF-deficient mice [39]. BAFF signaling through BAFF-R governs transitional differentiation and the survival of mature B cells [34, 36]. BAFF is biologically active in a soluble form after being cleaved by furin at the N-terminus of the TNF homology domain [35]. *In vitro* studies on B cells have shown that recombinant soluble BAFF can maintain the survival of mouse peripheral blood B cells and induce their proliferation [40–42]. Soluble BAFF has also been proven to provide a survival signal to induce murine B cell expansion and to protect activated B cells from apoptosis [40–46].

In this study, we attempted to expand human B cells *in vitro* by using both BAFF and CD40L with an aim to expand these cells while maintaining their antigen-presenting ability. We first established a 293T-derived cell line that could simultaneously express the human costimulatory molecule CD40L and the soluble anti-apoptotic cytokine BAFF. A long-term co-culture model based on the co-operation between CD40L and soluble BAFF enabled the growth of a larger number of human B cells *in vitro* than that achieved with the use of feeder cells expressing only CD40L. The expression of the co-stimulatory molecules, CD80 and CD86, on the co-cultured B cells also significantly increased, leading to an enhanced antigen-presenting function. The educated CTLs exerted potent anti-tumor and anti-viral activity. Thus, our approach could generate an almost unlimited source of antigen-specific APCs for use in adoptive immunotherapies.

## RESULTS

### The 293T-CD40L-sBAFF cell line significantly stimulates B cell growth

**Figure 1:**
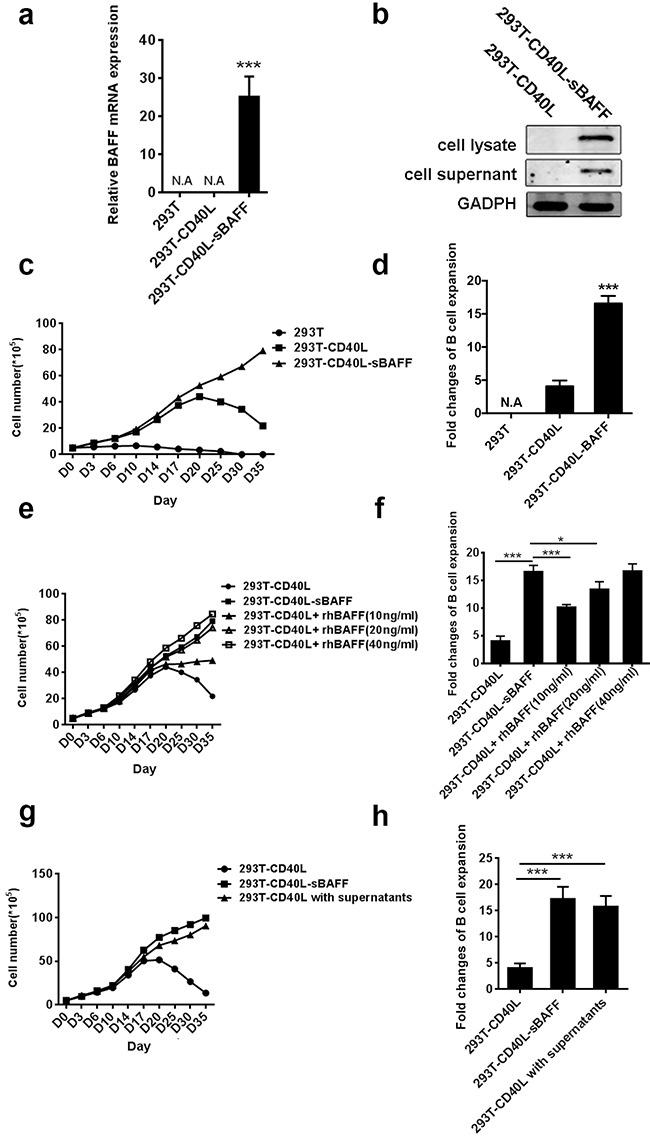
The 293T-CD40L-sBAFF cell line had the better potential to stimulate B cell growth **a.** BAFF mRNA expression was confirmed by quantitative RT-PCR. **b.** Western-blotting confirmed the presence of Flag-tagged soluble BAFF in the cell lysate and the supernatant. **c-h.** B cells from multiple healthy adult donors were co-cultured with the irradiated 293T, 293T-CD40L or 293T-CD40L-sBAFF feeder cells in the presence of cytokine cocktails as well as the indicated treatments. The B cells were harvested every 3-5 days and the cell numbers excluding trypan blue-positive dead cells were counted, with the average measurements for three donors and their standard deviations over time are shown. (c) Expansion patterns and (d) The B-cell expansion change in the presence of 293T-CD40L-sBAFF cells, 293T-CD40L cells, or 293T cells at day 35. (e) Expansion patterns and (f) expansion fold change of B-cells in the presence of 293T-CD40L-sBAFF cells, or 293T-CD40L cells with varying concentrations of recombinant human BAFF (rhBAFF) at day 35. (g) Expansion patterns and (h) expansion fold change of CD40L-B cells with the filtered supernatants of 293T-CD40L-sBAFF cells at day 35. Data represent mean ± SD (error bars) for a representative experiment of n = 3 independent experiments. The paired t-test and one-way ANOVA were used. P < 0.05 indicates statistically significance difference. * indicates P < 0.05; ** indicates P < 0.01; *** indicates P < 0.001.

In order to expand xenoantigen-free B cells effectively, a stable human HEK293T cell line expressing membrane-bound CD40L, named 293T-CD40L, was generated as described previously [47]. The expression of membrane-bound CD40L was stable for at least 3 months (Supplementary Figure S1). Although the combination of IL-4 and membrane-bound CD40L is essential for inducing B-cell expansion *in vitro* [48], activated B cells are prone to apoptosis [29, 30], which may limit long-term B cell expansion in cell culture. Since BAFF can induce murine B cell proliferation and co-stimulate cells in the presence of CD40L [35], a human HEK293T cell line expressing both membrane-bound CD40L and soluble human BAFF, named 293T-CD40L-sBAFF, was generated to increase the survival of the B cells. The expression of BAFF in the 293T-CD40L-sBAFF cell line was confirmed (Figure 1a). To assess the production of soluble BAFF, the supernatants of the 293T-CD40L-sBAFF cell cultures were collected and the proteins were concentrated and analyzed by western blot (Figure 1b).

After 3 weeks of stimulation according to previously described protocols [15, 17], the B cells activated with the 293T-CD40L cell line, which are referred to as CD40L-B cells hereafter, increased approximately 9-fold in number as compared to the B cells co-cultured with the 293T cell line (referred to as 293T-B) (Supplementary Figure S2). However, during the expansion process, a substantial decline in CD40L-B cell expansion was observed after 3 weeks of stimulation with the 293T-CD40L cell line. In contrast, the expansion patterns of the B cells activated with the 293T-CD40L-sBAFF cell line, which are referred to as the CD40L-sBAFF-B cells hereafter, were similar to those of the CD40L-B cells in the first week, but showed significant difference thereafter. After 2 weeks, the expansion of the CD40L-sBAFF-B cells was higher than that of the CD40L-B cells (Figure 1c). We also observed a sustained increase in the expansion of the CD40L-sBAFF-B cells after the third week. After 35 days, the number of CD40L-sBAFF-B cells increased to approximately 21-fold. However, the number of CD40L-B cells showed less than 7-fold increase (Figure 1d). To assess the activity of BAFF produced by the 293T-CD40L-sBAFF cells (sBAFF), we compared it to that of an equal density of 293T-CD40L-cells with varying concentrations of commercially available GMP-grade recombinant human BAFF (rhBAFF). In the presence of cytokine cocktails, the efficiency of B cell expansion by sBAFF was higher than that by rhBAFF at a 10 ng/ml concentration, which is routinely used in mouse and human *in vitro* B cell cultures [49, 50] (Figures 1e, 1f). However, the increased concentrations of rhBAFF led to an increase in B cell expansion and eventually reached a level similar to that observed in the case of sBAFF. Alternatively, to ensure that the increase in amplification efficiency by the 293T-CD40L-sBAFF cells was due to the secretion of soluble BAFF, the B cells were co-cultured with the 293T-CD40L cell line, and the filtered supernatants of the culture of the 293T-CD40L-sBAFF cells. The result indicated that the supernatant of the 293T-CD40L-sBAFF cell culture indeed supported B cell expansion in the presence of the 293T-CD40L cells (Figures 1g, 1h). Collectively, our data indicated that the 293T-CD40L-sBAFF cell line supported B cell growth more efficiently and economically and for a much longer period.

**Figure 2:**
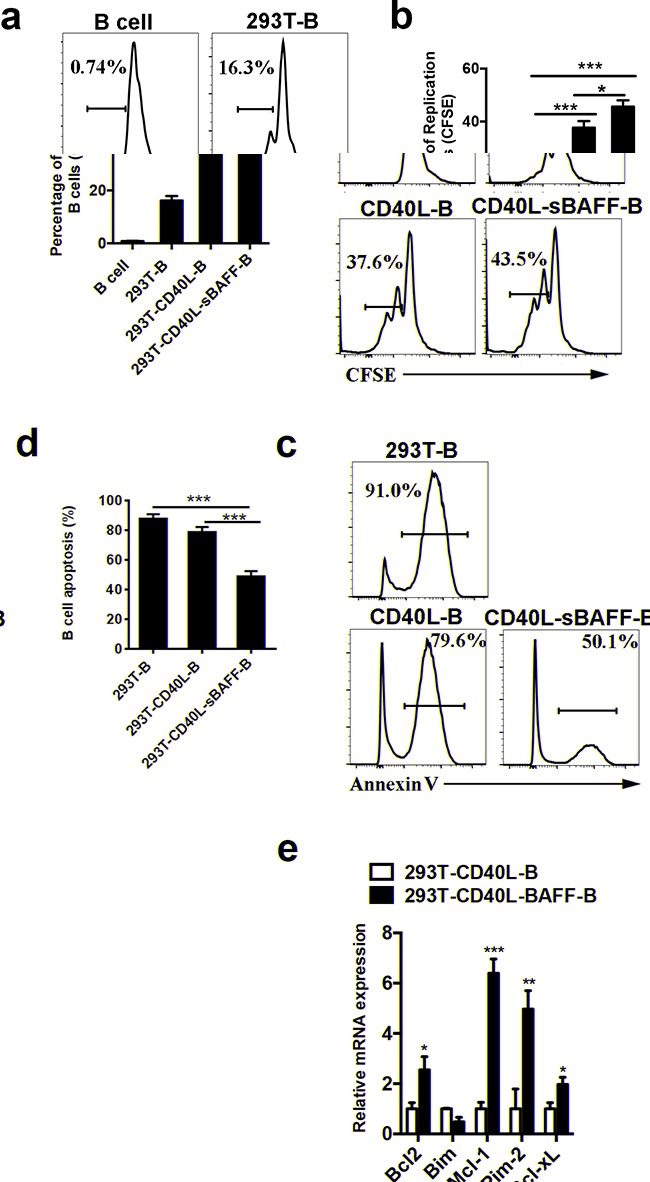
The 293T-CD40L-sBAFF cell line had the capacity to induce B cell growth and prevent apoptosis **a, b.** Cell division of 293T-B cells, CD40L-B cells and CD40L-sBAFF-B cells were measured by CFSE dilution with flow cytometry analysis, in comparison with non-activated cells (B cells) on day 7 after co-culture with feeder cells. **c, d.** Cell apoptosis of 293T-B cells, CD40L-B cells and CD40L-sBAFF-B cells were measured by Annexin V staining by flow cytometry on day 20 after co-culture with feeder cells. **e.** The mRNA expression of pro-apototic and anti-apoptotic factors in B cells. Data represent mean ± SD (error bars) for a representative experiment of 4 independent experiments with 4 healthy donors. The paired t-test and one-way ANOVA were used. P < 0.05 indicates statistically significance difference. * indicates P < 0.05; ** indicates P < 0.01; *** indicates P < 0.001.

The expansion of B cells was further verified using the CFSE assay. Analysis of B cell expansion by CFSE dilution revealed that the 293T-CD40L-sBAFF cell line was more efficient than the 293T-CD40L cell line in inducing B cell proliferation (Figures 2a, 2b). To assess whether the 293T-CD40L-sBAFF cells induced better survival of the B cells, the B cells in the cell culture at day 20 were harvested, and cell apoptosis was measured by Annexin V/PI staining. Apoptosis was significantly lower in the CD40L-sBAFF-B cells than in the CD40L-B cells (Figures 2c, 2d), thus supporting our hypothesis that the prolonged culture time of the CD40L-sBAFF-B cells was indeed due to BAFF-mediated B cell survival and B cell proliferation. To further verify if the apoptosis of CD40L-sBAFF-B cells is blocked by BAFF-mediated pathways, we examined the effect of co-culture on various components in the pathways leading to BCR-induced cell death. CD40L-sBAFF-B cells expressed higher levels of anti-apoptotic factors Mcl-1 and Pim-2 and lower levels of proaptotic factors Bim, all of which have been reported to play a role in BAFF-mediated B cell survival [25, 32, 51] (Figure 2e). Upregulation of two anti-apoptotic Bcl-2 family members in the NF-κB pathway (Bcl-2 and Bcl-xL) was also observed (Figure 2e).

### CD40L-sBAFF-B cells had enhanced antigen-presentation abilities

**Figure 3:**
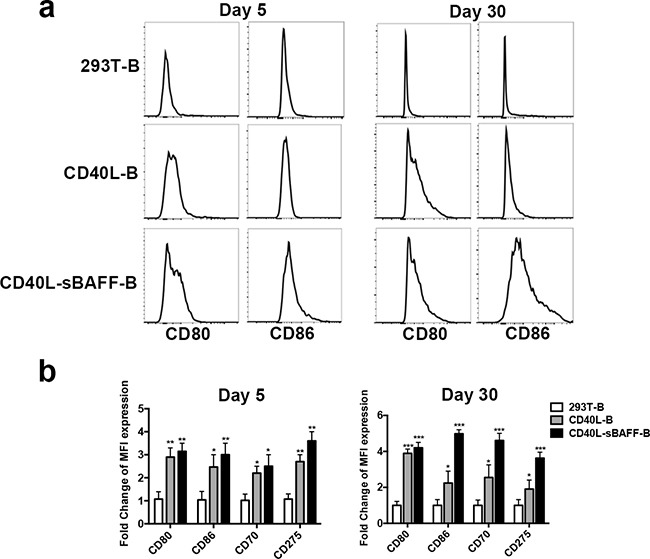
The co-culture with 293T-CD40L-sBAFF enhanced the expression of T co-stimulatory molecules on B lymphocytes On day 5 and day 30 after co-culturing, 293T-B cells, CD40L-B cells and CD40L-sBAFF-B cells were harvested. **a.** CD80 and CD86 expression were measured by flow cytometry. **b.** MFI expression fold changes of CD80, CD86, CD70, and CD275 on CD40L-B and CD40L-sBAFF-B cells, in comparison with that on 293T-B cells.

Since sufficient expression of costimulatory molecules closely correlates with APC function [15, 17, 52], a phenotypic analysis of cell surface markers on activated B cells was performed. We found that both the CD40L-B and CD40L-sBAFF-B cells had higher CD80 and CD86 expression than the controls on both day 5 and day 30 (Figures 3a, 3b). Interestingly, the CD40L-sBAFF-B cells showed significantly higher CD86 expression even compared to the CD40L-B cells on day 30. Moreover, the expression of other costimulatory molecules such as CD70 (CD27L) and CD275 (ICOS L) was also increased in the CD40L-sBAFF-B cells (Figure 3b).

**Figure 4:**
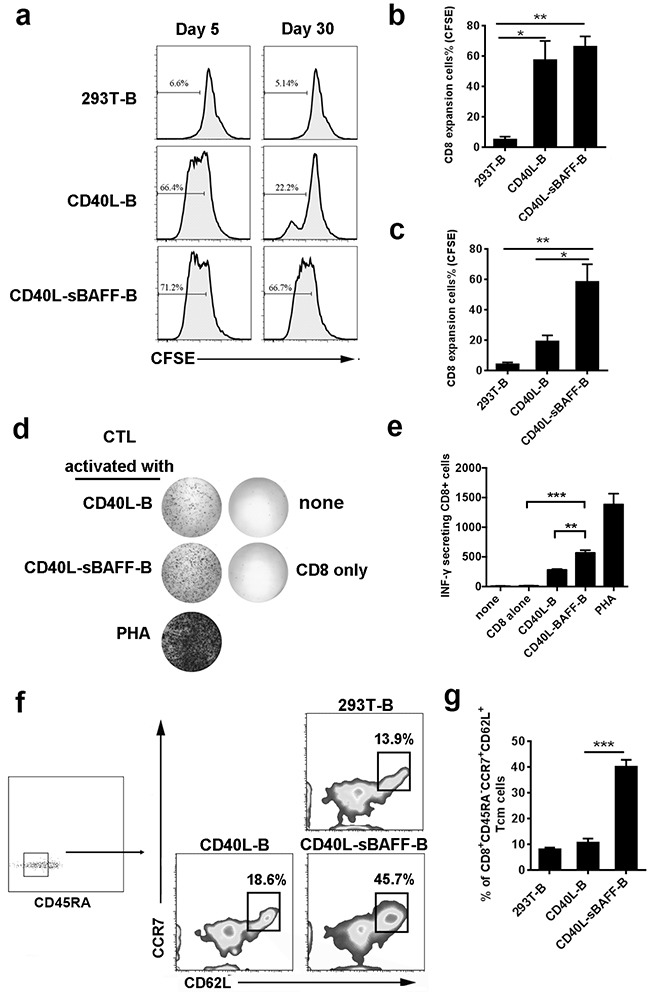
CD40L-sBAFF-B cells were of enhanced antigen-presentation capacities On day 5 and day 30 after co-culturing, 293T-B cells, CD40L-B cells and CD40L-sBAFF-B cells were harvested. **a-c.** B cells were co-cultured at an optimal ratio of 3:1 with CFSE-stained autologous CD8^+^ T cells. Cell divisions of CD8^+^ T cells were measured by CFSE dilution. **d-g.** B lymphocytes were cultured for 30 days, and then harvested, irradiated, washed, and co-cultured with purified autologous CFSE-labeled CD8^+^ T lymphocytes at an E: T ratio of 1:3 for one week. (d, e) Production of IFN-γ was measured by ELISPOT assay. (f, g) A representative profile indicating the percentage of CD8^+^ CD45RA^−^ CD62L^+^ CCR7^+^ central memory T lymphocytes after co-cultured with B lymphocytes. Data represent mean ± SD (error bars) for a representative experiment of three independent experiments. The paired t-test was used. P < 0.05 indicates statistically significance difference. * indicates P < 0.05; ** indicates P < 0.01; *** indicates P < 0.001.

The mixed lymphocyte reaction (MLR) assay was conducted to determine if the increased expression of costimulatory molecules on the CD40L-sBAFF-B cells enhanced the antigen-presenting ability of these cells [15]. The B cells were irradiated on days 5 and 30 and then co-cultured at an optimal ratio of 3:1 with CFSE-stained autologous CD8^+^ T cells. CD8^+^ T cell proliferation was measured by flow cytometry on the 7th day after co-culture. As expected, both the CD40L-B cells and CD40L-sBAFF-B cells irradiated on day 5 induced the proliferation of autologous CD8^+^ T cells (Figures 4a, 4b). The CD40L-sBAFF-B cells maintained their ability to stimulate the proliferation of CD8^+^ T cells even after expansion for 30 days, while the CD40L-B cells showed almost a complete loss of antigen-presenting ability at the same time (Figures 4a, 4c). Moreover, IFN-γ secretion from the CD8^+^ T cells generated using CpG-primed CD40L-B was much lesser than that from the CD40L-sBAFF-B cells (Figures 4d, 4e), which also supported our hypothesis that the CD40L-BAFF-B cells induced more production of functional CD8^+^ T cells than did the CD40L-B cells after culture for 30 days. Moreover, we observed that the expanded T cells exhibited an increased proportion of CD45RA^−^ CD62L^+^ CCR7^+^ central memory (Tcm) phenotype when co-cultured with CD40L-sBAFF-B cells (Figures 4f, 4g), which is encouraging given the significant role played by CD8^+^ Tcm cells in immunotherapy [53].

### CD40L-sBAFF-B cells pulsed with the HIV-1 peptide provided an effective source of APCs to generate HIV-1 antigen-specific CTLs

**Figure 5:**
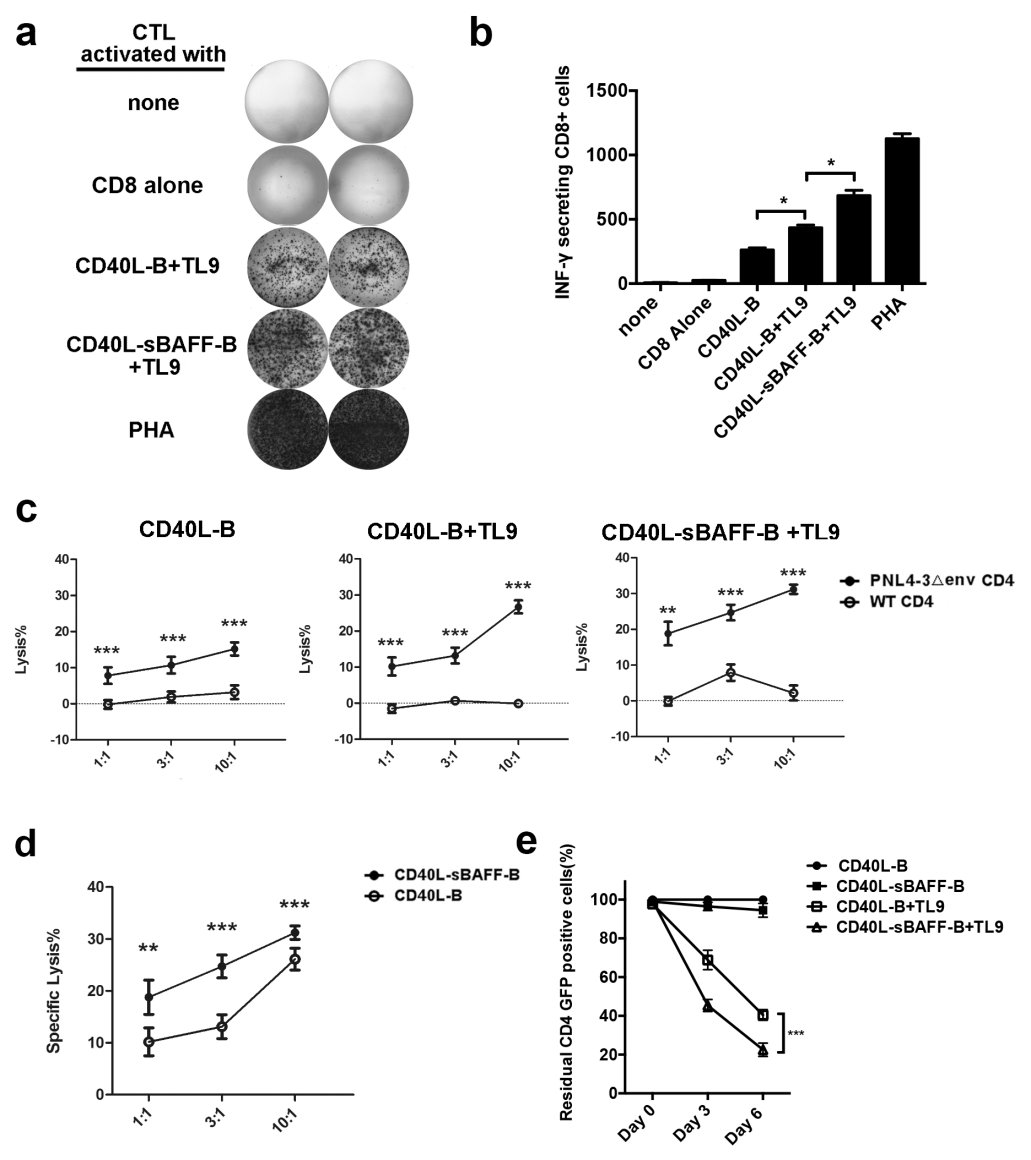
CD40L-sBAFF-B cells pulsed with TL-9 provided an effective APC source to generate HIV-1-specific CTLs At day 5 after co-culturing, CD40L-B cells and CD40L-sBAFF-B cells were harvested and incubated with peptide TL9 (20 ng/ml) at 37°C for 12 hours. Then autologous CD8^+^ T cells were co-cultured at a T: B ratio of 3:1 for 7 days with these irradiated B-cells. On day 12, CD8^+^ T lymphocytes were harvested, washed, and restimulated with fresh TL9-pulsed B cells and IL-2. This was repeated on days 19 and 26. After that, CTLs were co-cultured with HIV-1_NL4-3□env-EGFP_-infected primary CD4^+^ T cells as target cells. **a, b.** Representative results of IFN-γ production produced by the educated CTLs. The IFN-γ production was measured by ELISPOT assay. **c, d.** The educated HIV-1-specific CTLs were mixed with HIV-1_NL4-□env-EGFP_-infected primary CD4^+^ T cells as target cells at different E: T ratios. The cell lysis rate was measured with LDH-release assay. **e.** The calculation of survived HIV-1-infected CD4^+^ GFP^+^ cells after co-culture with the educated CD8^+^ T cells. The CD8 T-cells were educated with various B-cells. Then HIV-1-infected CD4^+^ GFP^+^ cells were mixed with the CD8 T-cells at E:T ratio of 3:1 in the presence of IL-2 for 6 days. The proportion of survived GFP^+^ cells was measured with FACS analysis. The survived GFP^+^ cells mixed with the CD8^+^ T-cells generated with TL9 unpulsed CD40L-B cells at day 0 were set as 100%. Data represent mean ± SD (error bars) for a representative experiment of three independent experiments. The student t-test and one-way ANOVA were used. P < 0.05 indicates statistically significance difference. * indicates P < 0.05; ** indicates P < 0.01; *** indicates P < 0.001.

Since the expression of CD80, CD86, CD70, and CD275 on the surface of the CD40L-sBAFF-B cells was significantly increased after co-culture with feeder cells, we hypothesized that these cells could help enhance the antigen-presenting ability. To test this hypothesis, HIV-1-derived peptides were used to pulse the B cells. We chose an immunodominant p24 Gag epitope TPQDLNTML (TL9; residues 180-188), which is among the most frequently recognized conserved epitopes in HIV-1-infected individuals and which exists in the HIV-1_NL4-3_-Δenv-EGFP sequence, to generate HIV-1-specific CTLs [54]. The function of the autologous CD8^+^ T cells, after three rounds of co-culture with TL9-pulsed CD40L-B or CD40L-sBAFF-B cells, was tested using the IFN-γ ELISPOT assay. The results showed that the TL9-pulsed CD40L-sBAFF-B cells generated a much higher number of IFN-γ–secreting CD8^+^ T cells than did the CD40L-B cells (Figures 5a, 5b).

To further evaluate the antiviral activity of the B cell-educated CTLs, we used an LDH release assay to test if they could kill HIV-1-infected CD4^+^ T cells. In order to test the antiviral activities of the CTLs educated using the TL-9-pulsed B cells, GFP^+^ HIV-1-infected CD4^+^ T cells were generated as the target cells by infecting the isolated primary CD4 T-cells with HIV-1_NL4-3_Δenv-EGFP/VSV pseudotyped viruses, followed by sorting of the GFP^+^ cells using FACS. The B cell-educated CTLs were then mixed with the target cells at different effector to target ratios. We found that the CTLs showed antigen-specific cytotoxicity against target cells (Figure 5c). The CTLs educated using the TL9-pulsed CD40L-sBAFF-B cells showed enhanced antigen-specific cytotoxicity against the target cells (Figure 5d), indicating that the CD40L-sBAFF-B cells indeed showed a higher efficiency of inducing antigen-presenting abilities. It is important to note that the cytotoxicity was mediated by HIV-1-specific CTLs, since the lysis of uninfected CD4^+^ T cells was not observed. Alternatively, the target cells were co-cultured with CTLs at an effector to target ratio of 3:1. The proportion of GFP^+^ cells, which represent the HIV-1-infected cells, was analyzed using FACS every 3 days to observe cell survival (Figure 5e). We found that the proportion of GFP^+^ cells decreased significantly in the presence of autologous CTLs educated using TL9-pulsed B cells. The CTLs educated using the CD40L-sBAFF-B cells formed the lowest proportion of residual GFP^+^ cells. Taken together, these results indicate that the CD40L-sBAFF-B cells provided a novel strategy to educate HIV-1-specific CTLs, which can effectively kill HIV-1-infected primary CD4 T cells.

### Autologous CTLs educated using CD40L-sBAFF-B cells enhanced the capability to eliminate reactivated HIV-1-infected CD4+ T cells isolated from HIV-1-infected individuals receiving suppressive ART

**Figure 6:**
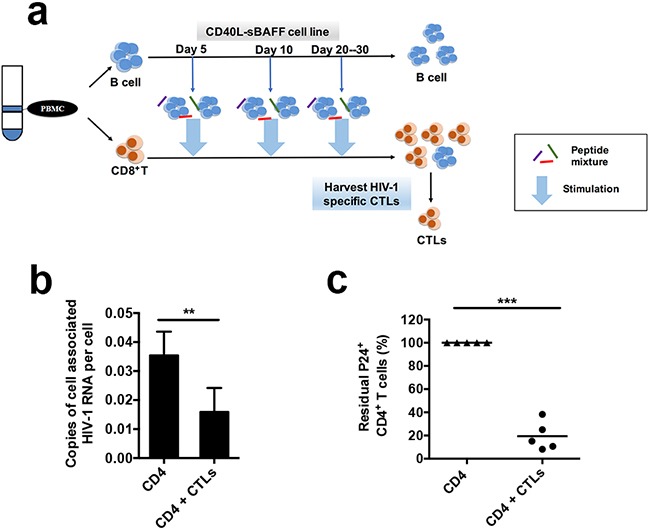
The autologous CTLs educated by CD40L-sBAFF-B cells enhanced the capability to eliminate the reactivated HIV-1-infected CD4^+^ T cells isolated from HIV-1-infected individuals receiving suppressive cART **a.** The manufacturing strategy of HIV-1 antigen-specific CTLs. The PBMCs were isolated from ten whole blood samples of HIV-1-infected individuals receiving suppressive cART. B cells were then isolated and co-cultured with the 293T-CD40L-sBAFF cell line in the presence of cytokine cocktails. The activated CD40L-sBAFF-B cells were pulsed with PepMixes containing WF9, TL9, TP9, HA9, and PY9 as described. Then, autologous CD8^+^ T cells isolated from remaining PBMCs co-cultured at a T: B ratio of 3:1 with irradiated CD40L-sBAFF-B cells and 20 ng/ml IL-2. T cells underwent three weekly stimulations by co-culture with autologous, PepMix-pulsed B cells. **b.** HIV-1-infected individuals CD4^+^ T cells were isolated, stimulated with PHA and IL-2 overnight. PepMix-pulsed B cells-educated autologous CD8^+^ T cells were added into the culture of CD4^+^ T cells at the effector-to-target ratio 3:1 in the presence of T-20 to prevent further rounds of viral replication. After 5 days of co-culture, the cell-associated HIV-1 gag RNA was quantified by qRT-PCR. **c.** The fraction of residual p24^+^ CD4^+^ T cells was measured and normalized to the control culture without CTLs. Data represent mean ± SD (error bars). The paired t-test was used. P < 0.05 indicates statistically significance difference. * indicates P < 0.05; ** indicates P < 0.01; *** indicates P < 0.001.

A broad CTL response has been used previously to clear HIV-1 latently-infected cells by utilizing a peptide mixture [7]. However, the generation of a population of HIV-1 antigen-loaded APCs is still required to elicit strong CTL responses for anti-HIV-1 immunotherapy [55]. To generate a sufficient amount of high-quality CTLs from HIV-1-infected individuals, we tried to use the HIV-1 peptide-pulsed CD40L-sBAFF-B cells as APCs. To this end, the B cells isolated from the PBMCs of patients on suppressive combined antiretroviral therapy (cART) were co-cultured with 293T-CD40L-sBAFF feeder cells and pulsed with a Gag peptide mixture, which can induce broad CTL responses to clear HIV-1 latently-infected cells without the escaped mutations [7]. After two more rounds of stimulation with the HIV-1 peptide-pulsed CD40L-sBAFF-B cells, we obtained the *ex vivo* expanded HIV-1-specific broad CTLs (Figure 6a). Autologous CD4^+^ T cells reactivated using PHA were then prepared as reported previously [7], followed by co-culture with HIV-1-specific CTLs in the presence of the HIV-1 entrance inhibitor, enfuvirtide (T-20) to prevent further infections. After 5–7 days, the cells were harvested, and viral expression was indicated by cell-associated HIV-1 RNA levels. The CTLs generated using CD40L-sBAFF-B cells pulsed with the Gag peptide mixture induced a significant decrease in cell-associated HIV-1 RNA expression in the reactivated CD4 T-lymphocytes, indicating that they could efficiently kill the autologous infected CD4^+^ T cells (Figure 6b). To further evaluate HIV-1 clearance by the B cell-educated CTLs, the cells were also analyzed using FACS to detect intracellular HIV-1 p24 expression. FACS revealed a remarkable decrease in intracellular p24 expression after co-culture with the educated CTLs (Figure 6c). Together, these results indicated that the B-lymphocytes isolated from HIV-1-infected individuals receiving suppressive ART, after education with 293T-CD40L-sBAFF-cells and pulsing with a Gag peptide mixture, can induce a powerful broad CTL response to clear autologous CD4 T-lymphocytes reactivated from viral latency, and therefore, indicate a new approach for the “shock and kill” strategy for eradicating HIV-1 reservoirs.

### CTLs educated using NY-ESO-1-pulsed CD40L-sBAFF-B cells effectively inhibited the growth of A375 tumor xenografts *in vivo*

**Figure 7:**
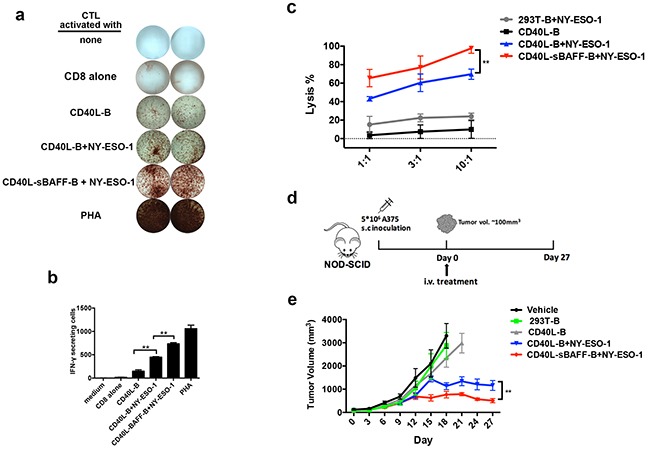
*In vivo* efficacy of autologous CTLs educated by CD40L-sBAFF-B cells pulsed with NY-ESO-1 in NOG xenograft models In order to present tumor-antigen to CD8^+^ T cells, B cells were incubated with NY-ESO-1 (20 ng/ml) for 12 hours, followed by irradiation and co-culture with autologous CD8^+^ T cells at a T: B ratio of 3:1. After a week, CD8^+^ T lymphocytes were harvested, washed and co-cultured with B cells for another 7 days. **a, b.** Representative results of IFN-γ production produced by the B cell-educated CTLs. The IFN-γ production was measured by ELISPOT assay. **c.** The B cell-educated CTLs were mixed with the NY-ESO-1 expressing melanoma cell line A375 at different E: T ratios. The cell lysis rate was measured with LDH-release assay. **d.** The strategy of *in vivo* experiment: NOD-SCID mice were engrafted with A375 melanoma cells (5 × 10^6^) and randomly allocated to five groups which were designated as treatment or control group. Mice were intravenously injected with autologous CTLs educated by NY-ESO-1 pulsed 293T-B cells, NY-ESO-1 pulsed CD40L-B cells, NY-ESO-1 pulsed CD40L-sBAFF-B cells or unpulsed CD40L-B cells. N = 8 mice per group. **e.** Injection of tumor-specific CD8^+^ T cells educated by NY-ESO-1 pulsed CD40L-BAFF-B cells effectively inhibited the growth of A375 tumor xenografts in mice. P < 0.01 after twenty days. Mann-Whitney U-test was used.

Melanoma is considered one of the best examples of an immunogenic tumor. Adoptive immunotherapy against melanoma has shown remarkable long-term results [56, 57]. In this study, to further confirm if the CTLs educated using CD40L-sBAFF-B cells exhibited better anti-tumor activities, a cancer-testis antigen NY-ESO-1 was utilized to pulse the CD40L-sBAFF-B cells in order to generate tumor-specific CTLs. The CD8^+^ T cells generated using the NY-ESO-1 pulsed CD40L-sBAFF-B cells showed the highest IFN-γ secretion, which was confirmed using the ELISPOT assay (Figure 7a). Moreover, the CTLs generated using the NY-ESO-1-pulsed B cells showed a strong cytotoxicity against A375 cells. In contrast, no cytotoxicity was observed against the control targets. Peptide-pulsed CD40L-sBAFF-B cells also exhibited an enhanced lysis ratio (almost 100%) against the target cells (Figure 7b).

Although activated B cells have been identified as alternative APCs for cancer immunotherapy in a series of *in vitro* experiments, their functionalities in cancer immunotherapy still need to be further analyzed due to the lack of evidence from *in vivo* experiments [8, 55]. To verify that CD40L-sBAFF-B cells were efficient *in vivo*, we took advantage of an extensively studied melanoma xenograft model system. When tumors reached a size of approximately 100 to 150 mm^3^, mice were randomly allocated to five groups designated as various treatment or control groups. Then, autologous CTLs educated using the NY-ESO-1-pulsed 293T-B cells, NY-ESO-1-pulsed CD40L-B cells, NY-ESO-1-pulsed CD40L-sBAFF-B cells, or unpulsed CD40L-B cells were intravenously injected into the mice (Figure 7d). Tumor size was measured using calipers every 3 days and tumor volume was estimated. Over the 30 days of the study period, adoptive transfer of autologous CTLs educated using NY-ESO-1-pulsed CD40L-B cells or NY-ESO-1-pulsed CD40L-sBAFF-B cells effectively inhibited the growth of A375 tumor xenografts in the mice. Moreover, the NY-ESO-1-pulsed CD40L-sBAFF-B cells showed an enhanced capacity to generate potent A375-specific CD8^+^ T cells (Figure 7e). These in vivo data further indicated that CD40L-sBAFF-B cells were capable of generating more potent tumor antigen-specific CTL responses.

## DISCUSSION

Since Banchereau and collaborators reported that the culture of human B cells lasted several weeks with the use of the CD40L system, this culture system has been used to activate human B cells [58, 59]. However, the engagement of CD40L on B cells also made the cells more prone to apoptosis [29, 60], which was a significant obstacle for long-term B-cell expansion *in vitro* by shortening the co-culture time and affecting the cell state. To achieve higher expansion efficiency of human B-lymphocytes *in vitro*, we tried to develop a long-term co-culture model by introducing an anti-apoptosis cytokine, BAFF, into the CD40L system. Among the cytokines controlling lymphocyte survival, only members of the BAFF superfamily have yet been introduced into the CD40L-dominated culture system. BAFFs regulate humoral immunity by controlling B lymphocyte differentiation [61], selection, and survival [37, 42, 43, 62, 63]. A lack of involvement of APRIL in B cell survival was indicated by a normal B cell compartment in a mouse expressing murine BCMA-Ig as a transgene [64]. Therefore, we introduced BAFF rather than APRIL into the CD40L-dominated culture system. During culture for 35 days, the number of live CD40L-sBAFF-B cells increased continuously; on the contrary, CD40L-B cells showed a decrease in their number after 3 weeks. Soluble GMP-level BAFF is expensive and not easily available, but our new system provides an economic and efficient method to increase the expansion efficiency and to prolong the culture time of human B cells.

Activated B cells can induce the activation and proliferation of antigen-specific CTLs for anti-tumor and anti-virus activities *in vitro* [15, 17, 65]. Since this process depends on the antigen-presenting capacity of the activated B cells, the results of phenotypic analysis of the cell surface molecules associated with the antigen-presenting function are considered reliable parameters [66]. We found that the 293T-CD40L-sBAFF-B cells, after long-term culture, showed significantly higher expression of CD86, a marker of antigen-presenting function. This result is consistent with previous reports suggesting that the interaction between BAFF and BCMA resulted in the up-regulation of surface molecules critical for antigen presentation through the NF-ĸB and JNK signaling pathways triggered by BCMA [67, 68]. The interaction between increased CD86 expression on B cells and CD28 on CD8 T-cells could induce stronger T cell stimulatory signals, which caused the T cells to stably secrete IL-2 and other cytokines and to induce T cell proliferation while maintaining T cell survival [69]. Our results indicate that the 293T-CD40L-sBAFF-B cells showed enhanced ability to stimulate the proliferation of CFSE-labeled autologous CD8^+^ T cells and increased CTL function, as indicated by the IFN-ELISPOT assay (Figure 4). This result confirmed that the addition of BAFF improved the antigen-presenting capacity of the B cells. Although it has been claimed previously that CD40L-B cells can expand antigen-experienced CD8^+^ T cells, which have memory-like function, and prime naive CD8^+^ T cells *in vitro* [15, 18], the subsets of CD8^+^ T cells after priming using autologous B cells were not analyzed. We observed that co-culture with CD40L-sBAFF-B cells increased the proportion of cells showing the Tcm phenotype with higher expression of CD62L and CCR7. This data is interesting since CTLs with a Tcm phenotype showed long-lasting memory T cell properties that enable superior persistence and expansion *in vivo* after adoptive transfer [53]. Thus, our new system can not only increase the expansion efficiency and prolong the culture time of human B cells but also enhance the antigen-presenting capacity of these cells.

cART suppresses HIV-1 replication to an undetectable level in the blood plasma and has been shown to increase the lifespan of HIV-1-infected individuals. However, in our previous studies, we confirmed that one major limitation of cART is that it fails to eradicate the latent HIV-1 reservoir [70]. Instead of a ‘sterilizing’ cure to completely eradicate a viral reservoir, we and other research groups have extensively discussed a more feasible strategy known as ‘functional cure’ [71–74]. This strategy involves long-term host-mediated control of viral replication and remission of the symptoms of HIV-1 infection in the absence of antiretroviral therapy. As latently-infected resting CD4^+^ T cells usually do not present the viral antigen, CD8^+^ T cells cannot recognize and destroy them; this makes it important to reactivate HIV-1 latency using various agents [7, 75]. This strategy is named as “shock.” To efficiently eradicate reactivated HIV-1-infected cells, a “kill” strategy is subsequently needed to rebuild a long-term anti-HIV-1 immune surveillance system. Efficient CTL immunotherapy requires the use of an appropriate target antigen and loading strategy, optimization of the interaction between the antigenic peptide and T cells, and prevention of negative regulatory elements [76]. Rebuilding immunity in patients by autologous adoptive transfer of HIV-1-specific CD8^+^ T cells is a feasible strategy for directly enhancing the CTL response of patients on cART [7, 8, 77]. Recent studies have shown that broad-spectrum CTLs have potent antiviral activity and can eliminate target cells both *in vitro* and in humanized mice [7, 8]. Applications using this broad-spectrum antigen-specific CTL response could be further developed for HIV-1 immunotherapy. However, it is important to generate a sufficient amount of highly efficient APCs for CTL immunotherapy.

In a recent study, DCs were used to expand HIV-1 CTLs [77]. As monocytes/DCs account for a small portion of the PBMCs and can only be poorly expanded *in vitro*, a genetically modified K562 cell line was used to substitute DCs in the subsequent two rounds of stimulation. However, the presence of the K562 tumor cell line in this method may pose a potential risk in clinic usage. Because the expanded B cells possess almost the same capacity to induce the expansion of CTLs as do monocyte-derived DCs [27, 62], they should be a better choice. In this study, we used CD40L-sBAFF-B cells expanded *in vitro* to educate a sufficient amount of HIV-1-specific CTLs. CD40L-sBAFF-B cells not only show better antigen-presenting capacity than CD40L-B cells, but can also be expanded exponentially over several weeks with high purity and without the loss of antigen-presenting function. thus, this system provides an almost unlimited source of cellular adjuvant to achieve sufficient expansion of CTLs in several rounds of stimulation [18]. In this study, we took an additional step to demonstrate that the B cells could be used as APCs to generate HIV-1-specific broad-spectrum CTLs in HIV-1-infected patients. The CTLs generated using our novel CD40L-sBAFF-B system showed potent capability to eradicate reactivated HIV-1-latently infected cells.

Moreover, our work indicates that CTLs educated using our novel CD40L-sBAFF-B system showed the ability to eradicate tumor xenografts in tumor-bearing mice. After education using NY-ESO-1-pulsed autologous CD40L-sBAFF-B cells, tumor-specific CTLs exhibited a potent capacity to kill cancer cells and inhibit tumor growth. Thus, our novel CD40L-sBAFF-B system indeed provides a way to generate an almost unlimited source of tumor-specific APCs for use in adoptive immunotherapies, which shows a specific benefit for frequent vaccinations in tumor-bearing hosts [8].

In summary, we not only excessively expanded human B-lymphocytes *ex vivo*, but also potently enhanced their antigen-presenting ability in a co-culture with both CD40L and BAFF. Our data indicate that the addition of BAFF in the CD40L-dominated system is a quite important. We believe that this convenient system, as an important part of adoptive transfer for CTL-mediated immunotherapy, will eventually find application in the clinic for anti-tumor or anti-chronic viral infection therapies.

## MATERIALS AND METHODS

### Ethics statement

This research was approved by the Ethics Review Board of the Eighth People's Hospital at Guangzhou (Guangzhou Infectious Disease Hospital) in China and the Ethics Review Board of Sun Yat-Sen University. HIV-1-infected individuals were recruited at The Eighth People's Hospital at Guangzhou and given written informed consent with approval of the Ethics Committees. Twenty ml of whole blood sample was obtained from HIV-1-infected individuals on suppressive combination antiviral therapy (cART). De-identified human peripheral blood mononuclear cells (PBMCs) from healthy blood donors were obtained from local blood bank. We did not have any interaction with these human subjects or protected information, and therefore no informed consent was required.

### Generation of a CD40L-expressing and the soluble BAFF-secreting 293T cell line

The 293T-CD40L cell line was established according to relevant reports [15, 17]. Briefly, the CD40L cDNA-based PCR product was digested with NheI and AgeI restriction endonucleases (New England Biolabs) and then ligated to corresponding sites of the lentiviral vector pcPPT-IRES-GFP. The resulting pcPPT-CD40L-IRES-EGFP vector was transfected together with psPAX and pMD2G into the HEK293T cell line with the calcium-phosphate precipitation method. The supernatants were harvested at 48 h or 72 h after transfection. The collected supernatant was then co-cultured with 70% confluent HEK293T cells. On the third day after infection, GFP positive cells were sorted with the SORP FacsAria II (BD). Several rounds of sorting were implemented before generating a stable feeder cell line with approximately 92% cells expressing CD40L. Alternatively, the sequence of soluble forms of BAFF was amplified from cDNA with forward primer (5′-CTACTAGCATGGCCGGTCAGGGTCCAGAA-3′) contains NheI site and reverse primer (5′-CATGCCATGCTCACTTGTCATCGTCATCCTTGTAATCCAGCAGTTTCAATGCAC-3′) contains AgeI site. The PCR product was digested with NheI and AgeI restriction endonucleases (New England Biolabs) and then ligated to corresponding sites of the lentiviral vector pcPPT-IRES-RFP. The resulting pcPPT-sBAFF-IRES-RFP was transfected together with psPAX and pMD2G into HEK293T cells. The supernatants were harvested at 48 h or 72 h after transfection. The collected supernatant was co-cultured with 70% confluent 293T-CD40L cells as described above. On the 6th day after infection, GFP and RFP double positive cells were sorted with the SORP FacsAria II (BD). Several rounds of sorting were performed before generating a stable feeder cell line with approximately 80% cells expressing full-length CD40L and soluble BAFF.

### Expansion of human B cells

The 293T-CD40L, 293T-CD40L-sBAFF and normal HEK293T cell lines were seeded on a 12-well plate at 5×10^5^ cells/well or 2×10^5^cells/well, as described previously [48]. To prepare feeder cells, the cells was irradiated (95 Gy) after one day of seeding and incubated at 37°C for 12 hours to confirm growth arrest of the cells. The optimal ratio of cell line to B lymphocytes was determined by co-culturing feeder cells: B cells at ratios from 3:1 to 1:3, while the optimal density of B lymphocytes was determined from 5×10^4^/well to 1×10^6^/well. Every 4 days, the growing B cells were harvested and centrifuged in a Ficoll-Paque density gradient, counted, and analyzed by flow cytometry. B-cell co-cultures were performed in 12-well plate in the conditioned Iscove's Modified Dubecco's Medium (IMDM, Gibco), supplemented with 10% heat-inactivated pooled human AB serum (Sigma), 100 U/ml of penicillin, and 100 U/ml of streptomycin (Hyclone), 2 μg/ml CpG-ODN2006/2219 (Invitrogen), 0.625 μg/ml CsA (Cyclosporine A) (Sigma), 50 μg/ml transferrin (Sigma), 10 ng/mL IL-2 (Peprotech,), 10 ng/ml IL-10, and 20 ng/mL IL-4 (Peprotech).

### Quantitative real-time RT-PCR analysis

For the measurement of mRNA level, total RNAs were isolated with TRIzol reagent (Invitrogen) and then subjected to cDNA synthesis using PrimeScript RT reagent Kit (Takara). Quantitative PCR was performed with SYBR Premix ExTaq II Kit (Takara) by using the CFX96 Real-Time System (Bio-Rad). The instructions of the manufacturer were followed. Quantification was normalized to mRNA levels of the housekeeping genes such as GAPDH or β-actin.

### Antibodies

Several antibodies were used for flow cytometry analysis: anti-CD19 (HIB19); anti-CD80 (L307.4); anti-CD86 (FUN-1); anti-CD8 (RPA-T8); anti-CD40L (24-31); anti-P24 (59768) anti-CCR7 (3D12); anti-CD62L (GREG56); anti-CD45RA (UCHL1). All of these antibodies were purchased from eBioscience except for both anti-CD62L (GREG56) and anti-CD45RA (UCHL1), which were from Biotechnology; anti-CD19, which was from BD; and anti-P24, which was from Santa Cruz. Biotechnology. Antibodies for western blotting were as follows: Flag antibody (MBL, M180-3), and GAPDH antibody (PeoteinTech, 10494-1-PI).

### Flow cytometry

Cells were collected and washed in PBS with 0.5% BSA. Then the single-cell suspensions were labeled on ice for 30 min with various antibodies. P24 positive CD4^+^ T cells were measured by intracellular p24 staining after fixation and permeabilization using cytofix-cytoperm (BD Biosciences). Flow cytometry was performed on Fortessa (BD) and were analyzed with FlowJo software.

### Western blot

Supernatants of 293T-CD40L or 293T-CD40L-sBAFF cells were harvested at 48 h after the cells were seeded in the plate. Part of them (200 μl) was mixed with 50 μl 5*SDS gel-loading buffer and boiled to denature proteins. Cells were lysed with lysis buffer [150 mM NaCl, 50 mM Tris-HCl (pH 7.5), 1 mM EDTA, 1% Triton X-100, and 0.5% NP-40]. Each lysate or denatured supernatant was separated by 15% SDS-PAGE and analyzed by immunoblotting using primary antibodies as described. The LI-COR Odyssey scanner was used to detect and quantify fluorescent signals as previously described.

### CFSE staining

The protocol described previously was followed with minor modifications [78]. Briefly, every 2×10^6^ B cells or CD8^+^ T cells were incubated with 100 μl PBS and 2 μl CFSE at 37°C. After 20 minutes, the reaction was terminated by adding ice-cold IMDM. Cells were centrifuged at 380 g for 20 minutes, followed by rinsing twice by PBS. Cell divisions were measured by FACS analysis.

### B cell apoptosis

The amplified B cells were collected at day 20, washed twice with Annexin binding buffer and incubated with 5 μl Annexin V-FITC and 5 μl propidium iodide for 15 min according to the manufacturer's protocol (Dōjindo Laboratories, AD10). The cell apoptosis was analyzed by FACS on Fortessa (BD).

### MLR assay

MLR was performed as described previously [15, 19]. After stimulation, B cells were irradiated (75 Gy) and plated in 96-well U-bottom plate at different cell densities. The CFSE-labeled T cells were co-cultured at a T: B ratio of 3:1 with irradiated B-cells and 20 ng/ml IL-2 after isolated from PBMCs. Half of the medium volume was substituted every 3-4 days and cytokines were added to the original concentration during the 7-day-long co-culture assay. Cells were then harvested after 7 days, and CFSE was measured by FACS analysis.

### Enzyme-linked immunospot (ELISPOT) assays

ELISPOT assays were performed with a commercially available human IFN-γ precoated ELISPOT kit (DAKEWE). The manufacturer's protocol was followed with minor modifications. Briefly, the purified CD8^+^ T cells (7.5×10^4^/well) were mixed with expanded B cells (5×10^3^ cells/well). CD8^+^ T cell were then incubated with target cells on precoated PVDF plates for 18 h. To visualize spots, streptavidin-HRP and substrate were added. Spots were enumerated using a CTL Immunospot S5 core analyzer, and the data were analyzed using CTL ImmunoSpot software (Cellular Technology).

### Peptide pulsing

The HIV-1 Gag CTL Epitope TL9 [54] (YMNGTMSQV, 180-188), WF9 (WASRELERF), TP9 (TPGPGVRYP), HA9 (HPVHAGPIA), PY9 (PPIPVGEIY) and tumor-specific NY-ESO-1 peptide (SLLMWITQC) were synthesized by Bambio (Xiamen, China). The CD40L-B cells or CD40L-sBAFF-B cells were loaded with peptides (20 ng/ml) at 37°C for 12 hours.

### In vitro HIV-1 infection

The CD4^+^ T cells from healthy donors were stimulated with anti-CD3 (1 μg/ml), anti-CD28 (1 μg/ml) and IL-2 (200 U/ml) in the conditioned RPMI1640 medium with 10% heat-inactivated fetal bovine serum and antibiotics for 2 days before infection. Activated CD4^+^T cells were then infected with HIV-1/VSV pseudo-typed viruses, which were generated from 293T cells transfected with pNL4-□env-EGFP and pVSVG. The virus amount used in infection was equivalent to 200 ng HIV-1 p24 antigen. The infection was performed by centrifugation of target CD4^+^ T cells with viruses at 2000 g for 1 h.

### CTL cytotoxicity assays

CTL cytotoxicity assays against target cells including normal CD4 cells or CD4 cells infected by NL4-□env-EGFP were tested by using LDH-release assay (CytoTox 96 Non-Radioactive Cytotoxicity Assay; Promega). The experiments were done in 96-well round-bottom plates according to the manufacturer's protocol. B-cells were isolated from the PBMCs with BD IMag™ anti-human CD19 particles, followed by co-culture with 293T-CD40L or 293T-CD40L-sBAFF in the presence of cytokine cocktail as described above. The remaining PBMCs were then cultured in the conditioned RPMI1640 medium supplemented with 10% heat-inactivated fetal bovine serum, antibiotics and IL-2. The B-cells co-cultured with 293T-CD40L cells or 293T-CD40L-sBAFF cells were harvested and then incubated with TL9 peptide (20 ng/ml) as described above. After irradiating at 75 Gy, and subsequently plating in 96-well U-bottom plate, the B-cells were co-cultured with autologous CD8 T-lymphocytes at a T: B ratio of 3:1 in the presence of 20 ng/ml IL-2. After 7 days, CTLs were harvested, washed, and re-stimulated with fresh activated B cells for 7 more days in the presence of IL-2. Afterwards, the target cells were plated in 96-well round plate at 1×10^4^ cells/well. CTLs were then added at an E: T ratio of 10:1, 3:1 or 1:1. The plate was then incubated at 37°C for 12 hours. Cytotoxicity was calculated by using the following formula:

### In vivo experiments

Human melanoma xenografts were established in 4-6-week old NOD-SCID mouse by subcutaneous inoculation of 5×10^6^ A375 cells to each mouse. When tumors achieved approximately 100 to 150 mm^3^, the mice were divided randomly into five groups. Autologous CTLs educated by NY-ESO-1 pulsed 293T-B cells, NY-ESO-1 pulsed CD40L-B cells, NY-ESO-1 pulsed CD40L-sBAFF-B cells or unpulsed CD40L-B cells were respectively injected into A375 tumor-bearing mice recipients i.v. through the lateral tail vein. Tumor size was measured using calipers every three days and tumor volume was estimated. Tumor xenografts were collected 35 days after adoptive transfer.

### Cell-associated HIV RNA quantification

The CD4 T-lymphocytes were isolated from patient samples which were described above. The RNA was then extracted from with TRIzol reagent (Nitrogen) and subjected to RT-PCR analysis. After reverse transcription, the cell-associated unspliced HIV-1 RNAs were determined with forward primer (5′-CTACTAGCATGGCCGGTCAGGGTCCAGAA-3′) and reverse primer (5′-CATGCCATGCTCACTTGTCATCGTCATCCTTGTAATCCAGCAGTTTCAATGCAC-3′). An *in vitro*-synthesized HIV-1 RNA, after quantification, was used as the external control for measuring cell-associated viral RNA [79]. The expression levels were calculated using the following equations: γ = 1546.5e-0.987Ct and Copies per cell = 1.6*1010*γ/(cell number), according to standard curve.

## SUPPLEMENTARY MATERIALS FIGURES


