# Supplementary material for: Efficient generation of antigen-specific CTLs by the BAFF-activated human B Lymphocytes as APCs: a novel approach for immunotherapy
Source: Oncotarget. 2016 Oct 21;7(47):77732–48. doi: 10.18632/oncotarget.12792 (PMC5363617; doi:10.18632/oncotarget.12792)
Supplement: Supplementary file 1 [file oncotarget-07-77732-s001.pdf]

Efficient generation of antigen-specific CTLs by the BAFF-activated human B lymphocytes as APCs: a novel approach for immunotherapy

SUPPLEMENTARY FIGURES

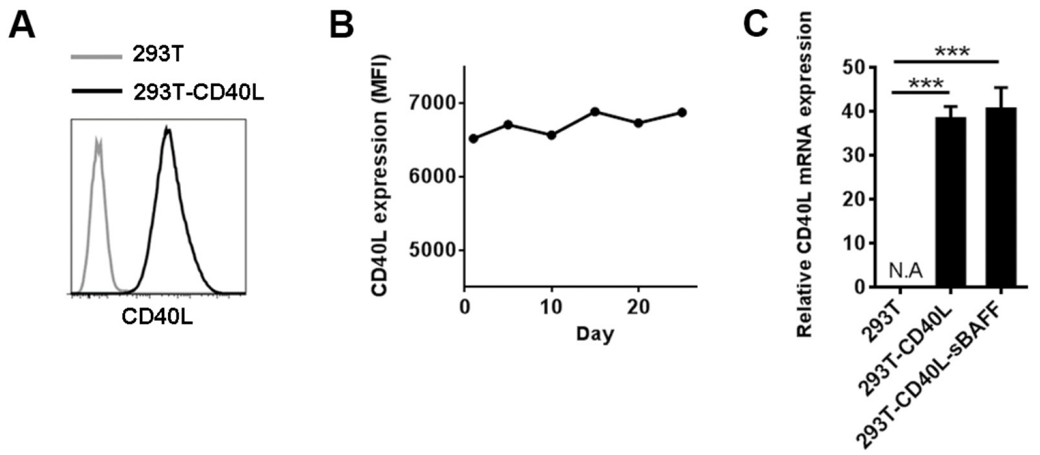

Supplementary Figure S1: The construction of 293T-CD40L and 293T-CD40L-sBAFF cell line. A. CD40L expression on the surface of HEK293T was measured by FACS analysis. **B.** Mean fluorescence intensities (MFI) of CD40L indicated that CD40L expression on cell surface remained stable during co-culture assays at different time points. **C.** CD40L mRNA expression in 293T-CD40L-sBAFF cell line was measured by quantitative RT-PCR. Data represent mean ± SEM (error bars). n = 3. P < 0.05 indicates statistically significance difference. *** indicates P < 0.001.

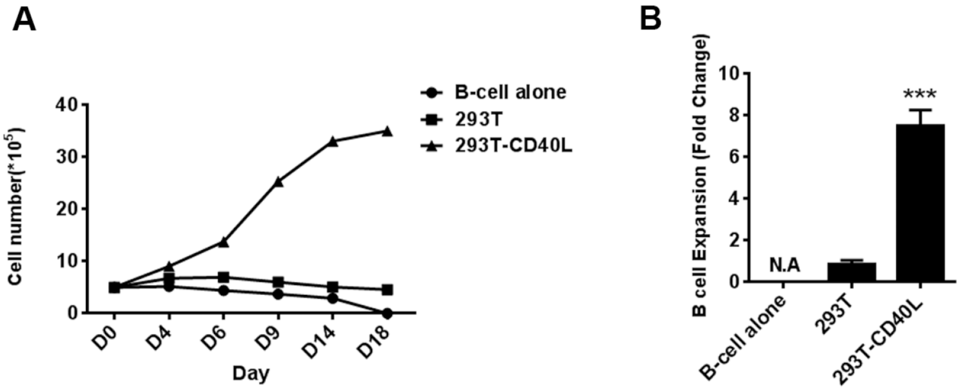

Supplementary Figure S2: The 293T-CD40L cell line had the capacity to stimulate B cells. A. Expansion patterns and **B.** expansion fold change of B cells stimulated with 293T-CD40L or HEK293T in the presence of cytokine cocktails (CpG-ODN2006, CsA, IL-4, IL-2, IL-10), compared with B cells co-cultured with 293T in the absence of cytokine cocktails. Data represent mean ± SEM (error bars). n = 3. P < 0.05 indicates statistically significance difference. *** indicates P < 0.001.
